# Unravelling HDL—Looking beyond the Cholesterol Surface to the Quality Within

**DOI:** 10.3390/ijms19071971

**Published:** 2018-07-06

**Authors:** Sarina Kajani, Sean Curley, Fiona C. McGillicuddy

**Affiliations:** Cardiometabolic Research Group, Diabetes Complications Research Centre, UCD Conway Institute, University College Dublin, Belfield, 4 Dublin, Ireland; sarina.kajani@ucdconnect.ie (S.K.); sean.curley@ucdconnect.ie (S.C.)

**Keywords:** HDL, cholesterol efflux capacity, cardiovascular disease, HDL lipidome, HDL proteome, HDL microRNA, HDL functions, inflammation

## Abstract

High-density lipoprotein (HDL) particles have experienced a turbulent decade of falling from grace with widespread demotion from the most-sought-after therapeutic target to reverse cardiovascular disease (CVD), to mere biomarker status. HDL is slowly emerging from these dark times due to the HDL flux hypothesis wherein measures of HDL cholesterol efflux capacity (CEC) are better predictors of reduced CVD risk than static HDL-cholesterol (HDL-C) levels. HDL particles are emulsions of metabolites, lipids, protein, and microRNA (miR) built on the backbone of Apolipoprotein A1 (ApoA1) that are growing in their complexity due to the higher sensitivity of the respective “omic” technologies. Our understanding of particle composition has increased dramatically within this era and has exposed how our understanding of these particles to date has been oversimplified. Elucidation of the HDL proteome coupled with the identification of specific miRs on HDL have highlighted the “hormonal” characteristics of HDL in that it carries and delivers messages systemically. HDL can dock to most peripheral cells via its receptors, including SR-B1, ABCA1, and ABCG1, which may be a critical step for facilitating HDL-to-cell communication. The composition of HDL particles is, in turn, altered in numerous disease states including diabetes, auto-immune disease, and CVD. The consequence of changes in composition, however, on subsequent biological activities of HDL is currently poorly understood and this is an important avenue for the field to explore in the future. Improving HDL particle quality as opposed to HDL quantity may, in turn, prove a more beneficial investment to reduce CVD risk.

## 1. HDL-C as a Traditional Biomarker for Cardiovascular Disease (CVD)

CVD is the leading cause of death worldwide and remains as such despite pharmacological interventions for numerous risk factors. Raising high-density lipoprotein cholesterol (HDL-C) levels pharmacologically has been a much-sought-after therapeutic strategy since its discovery in the 1960s and that of its inverse correlation to CVD events [1]. HDL-C is a robust, consistent, and independent predictor for CVD and, as such, has been included as a critical component in risk prediction by both the European and American Heart Associations [2,3].

The Framingham Heart Study in 1986 was the first landmark study to elucidate a connection between HDL-C and coronary heart disease (CHD) [4]. Other early studies found that increasing HDL-C by 1 mg/dL was associated with a 2–3% decreased risk of heart disease [5]. In parallel, the Emerging Risk Factors Collaboration included over 300,000 participants and found that the hazard ratio for CHD with HDL-C was 0.71 after adjustment for non-lipid risk factors [6]. Observational studies consistently revealed an inverse correlation between HDL-C and CVD leading to the hypothesis that raising HDL-C is protective against atherosclerosis [7,8]. Indeed, HDL particles are known to exert a number of important functions including anti-inflammatory, anti-oxidant, lipopolysaccharide (LPS)-sequestering, anti-thrombotic, and cholesterol-efflux-promoting functions which will be expanded upon later, all of which are thought to contribute to the anti-atherosclerotic effects of HDL. The ability of HDL to support cholesterol efflux from peripheral cells, including lipid-laden macrophages in atherosclerotic lesions, held great promise to promote regression of pre-established advanced lesions and, in turn, a resolute quest to identify targets that can increase HDL-C ensued. Such therapies were projected to be important companion therapies to statins wherein statins would limit further lesion progression, while HDL-raising therapy would reverse the established disease.

## 2. Raising HDL-C—The First Blow to the HDL Hypothesis

Cholesteryl ester transfer protein (CETP) mediates the transfer of cholesteryl ester from HDL to pro-atherogenic lipoprotein particles such as low-density lipoprotein (LDL) in exchange for triglycerides. Several population studies have revealed that genetic polymorphisms reducing CETP activity are associated with reduced CVD risk coincident with increased HDL-C and reduced LDL-C [9,10,11]. Furthermore, exceptional longevity and elevated HDL-C was evident within a population of Ashkenazi Jewish individuals who were homozygous for the 405 valine allele of CETP (loss of function mutation) [12]. CETP was therefore identified as a potential drug target and instigated the development of four major CETP inhibitors—Torcetrapib (Pfizer, New York, NY, USA), Dalcetrapib (Hoffmann–La Roche, Basel, Switzerland), Evacetrapib (Eli Lilly & Company, Indianapolis, IN, USA), and Anacetrapib (Merck, Kenilworth, NJ, USA).The Investigation of Lipid Level Management to Understand its Impact in Atherosclerotic Events (ILLUMINATE) clinical trial was the first major test for CETP inhibitors which entered a phase 3 clinical trial with 15,067 patients who were assigned to either Torcetrapib or placebo in a randomized, double-blinded manner. Despite a 72% increase in HDL-C and a near 25% decrease in LDL-C levels compared with placebo, Torcetrapib therapy resulted in increased risk of both CVD events (HR = 1.25) and death from any cause (HR = 1.58) which resulted in early termination of the trial (1–2 years duration). Potential causes of increased mortality were thought to be off-target effects unrelated to CETP inhibition and, in particular, increased blood pressure (+5.4 mmHg) through activation of the renin–angiotensin–aldosterone system [13].

The next CETP inhibitor to enter a phase 3 trial was Dalcetrapib which appeared to be devoid of serious blood-pressure-raising effects, giving a sense of cautious optimism within the CVD field. The Effects of the Cholesterol Ester Transfer Protein Inhibitor Dalcetrapib in Patients with Recent Acute Coronary Syndrome (dal-OUTCOMES) trial recruited 15,871 patients with recent acute coronary syndrome who were followed for a median of 31 months. While Dalcetrapib did modestly increase HDL-C levels (~30%) (with no changes in LDL-C), it did also increase C-reactive protein (CRP) compared with placebo. The trial was terminated on grounds of futility, as no reductions in recurrent cardiovascular events were observed [14]. A slight increase in blood pressure was also noted with Dalcetrapib (+0.6 mmHg), albeit to a much lesser extent than with Torcetrapib.

The Assessment of Clinical Effects of Cholesteryl Ester Transfer Protein Inhibition with Evacetrapib in Patients with a High Risk for Vascular Outcomes (ACCELERATE) trial followed on from dal-OUTCOMES. This multicenter, randomized, double-blind, placebo-controlled phase 3 trial sought to investigate the effect of Evacetrapib on cardiovascular outcomes in 12,092 patients with acute coronary disease or other vascular disease such as diabetes mellitus. Again, favorable effects on lipid parameters were established such as a 31% decrease in LDL-C and a striking 133% increase in HDL-C. Despite this, cardiovascular events rates were not lowered compared with placebo and after 26 months the trial was terminated early, again due to lack of efficacy [15].

The most recent and largest trial with CETP inhibitors was the Randomized Evaluation of the Effects of Anacetrapib through Lipid modification (REVEAL) trial which recruited over 30,000 adults with atherosclerotic disease and already on an intensive statin regimen who were assigned to Anacetrapib or placebo. The follow-up time on this trial was the longest for CETP inhibitors at 4 years. At the midpoint of this trial, as with the other trials, HDL-C was increased by 104% and LDL-C was reduced by 18% compared with placebo. Anacetrapib was the first CETP inhibitor that successfully reduced cardiovascular events (HR = 0.91), and reduced new-onset diabetes but, again, a small elevation in blood pressure was noted [16]. Furthermore, no differences in deaths from cardiovascular disease were noted (3.4% with Anacetrapib vs 3.7% with placebo). It was noted that Anacetrapib has a prolonged half-life with the drug accumulating within adipose tissue for several years despite cessation of treatment with Anacetrapib [17]. While adverse effects to date have not been cited, consequences of this side-effect will need to be monitored in patients of the REVEAL trial. Despite the successful reduction in cardiovascular events in Anacetrapib treated patients, this drug has not been brought forward by Merck for FDA approval, which likely signals the final nail in the coffin for targeting CETP therapeutically. CETP inhibition was seen as the Holy Grail in CVD drug development to mirror the HDL-C hypothesis. Given its consistent failures over time, one might argue the HDL-C hypothesis to be wrong. Within this review, we delve further beyond HDL-C levels and discuss quality as a better predeterminant of disease. Before the development of CETP inhibitors, niacin or nicotinic acid had been used for primary and secondary prevention of CVD for over 40 years [18,19]. The Coronary Drug Project aimed to investigate the efficacy of a number of lipid-mediating therapies, one of which was niacin treatment. In this trial, over 8000 men with previous myocardial infarction (MI) were recruited to a number of different pharmacological agents including niacin (*n* = 1119). Niacin reduced total serum cholesterol (−9.9%) and serum triglycerides (−19.4%) over 74 months and reduced rates of nonfatal MI (niacin, 12.6% vs placebo, 15.3%); however, this did not translate to reduced CHD mortality [19]. A 15-year follow-up study aimed at assessing long-term adverse effects of niacin was also conducted despite discontinuation of niacin at 74 months. There was a sustained benefit of niacin in terms of reduced total serum cholesterol (−10.1%) and reduced triglycerides (−26.9%) and, in turn, all-cause mortality in the niacin group (52%) was significantly lower than in the placebo group (58.2%). Deaths from CHD in the niacin group (36.5%) were also lower than in the placebo group (41.3%). It is important to note that these patients were statin-naïve [18]. These earlier studies conflict with more recent large clinical trials in 2011 and 2014, which found niacin treatment in combination with statin therapy did not provide clinical benefit over statins alone [20,21]. These discrepancies are likely due to lack of statin therapy in the original trial compared to latter trials where proof of clinical benefit above statin therapy was the sought-after endpoint.

The Atherothrombosis Intervention in Metabolic Syndrome with Low HDL/High Triglycerides and Impact on Global Health Outcomes (AIM-HIGH) trial, which recruited 3414 statin-treated CVD patients with low baseline levels of HDL-C, failed to demonstrate clinical benefit of extended-release niacin (ERN) over statin therapy alone. ERN therapy raised HDL-C from 35 mg/dL to 42 mg/dL and reduced triglycerides from 164 mg/dL to 122 mg/dL at the two-year point. This trial, however, was stopped after a follow-up period of 3 years due to lack of efficacy [21]. A serious adverse effect of niacin is vasocutaneous flushing which causes problems with compliance. The Heart Protection Study 2—Treatment of HDL to Reduce the Incidence of Vascular Events (HPS2-THRIVE) trial (*n* = 25,673) was designed to assess the effects of adding extended-release niacin in combination with laropiprant, a drug that reduces flushing, on cardiovascular outcomes in statin-treated high-risk patients with prior vascular disease [20]. Again, the ERN–laropiprant-treated group exhibited reductions in LDL-C (−10 mg/dL) and increases in HDL-C (+6 mg/dL) compared with placebo but no improvements in cardiovascular events were observed, similar to the AIM-HIGH trial outcomes. Furthermore, niacin–laropiprant treatment was associated with significantly more adverse events including increased diagnosis of diabetes. The lack of efficacy of HDL-C-raising therapies has caused many to question whether HDL particles are indeed bioactive or whether they are merely biomarkers of CVD—arguments that have been compounded by findings from genetic studies.

## 3. Lessons from Genome-wide Association Studies (GWAS)—The Second Setback for the HDL-C Hypothesis

The failure of CETP inhibitors was swiftly followed by a lack of association between novel, validated loci identified during a meta-analysis of 14 large-scale GWAS studies (22,233 CAD patients and 64,762 controls) and HDL-C levels [22]. A separate GWAS investigating loci that associate with blood lipid levels identified 95 loci across 100,000 participants of European descent. Genetic variants that associated with pro-atherogenic lipids/lipoproteins i.e., LDL-C, apoB, triglyceride-rich lipoproteins consistently associated with increased CVD risk; while genetic variants that impact specifically on HDL-C did not [23]. Another large-scale GWAS study (*n* = 188,577) investigated the genetic determinants of lipid levels and identified 157 loci that associate with lipid levels, with ~64 loci strongly associating with HDL-C levels. Trait-decreasing alleles of HDL-C were, in turn, associated with increased CAD risk (*p* = 0.02); however, when loci unique to lipid subpopulations were examined (LDL-C, 12 loci; triglycerides, 6 loci; and HDL-C, 14 loci), significance to CAD only remained for LDL-C. These large-scale GWAS studies are suggestive that genetically low HDL-C is not a determinant of CVD, which is in stark contrast to numerous epidemiology studies demonstrating the opposite.

A large part of our current understanding of HDL metabolism is due to Mendelian disorders of rare genetic variants which exert profound effects on the phenotype of interest. High HDL-C can be attributed to loss of function in CETP, endothelial lipase G (LIPG), and scavenger receptor class b member 1 (SCARB1), while mutations in ApoA1, ATP-binding cassette transporter A1 (ABCA1), and lecithin–cholesterol acyltransferase (LCAT) are associated with lower HDL-C [24,25]. While monogenic disorders are an incredibly valuable tool in investigating extreme phenotypes, they are rare and thus cannot explain population variability in HDL-C. Paradoxically, HDL-C levels that were specifically increased by loss of function of LIPG did not translate to decreased CVD risk [26]. Similar results were seen in the Copenhagen City Heart Study (CCHS, *n* = 10,281) and Copenhagen General Population Study (CGPS, *n* = 50,523) with a variant of LCAT (S208T, rs4986970, allele frequency 4%). This variant was associated with a 13% decrease in HDL-C but did not translate to increased incidence of MI. Interestingly, epidemiological analysis of the CCHS cohort demonstrated that a 13% reduction in HDL-C was associated with an 18% increase risk of MI [27]. These findings speak to the disconnect between genetically altered HDL-C and epidemiological observations on HDL-C. By contrast, similar studies done on common variants associated with LDL-C largely show a positive correlation to CVD [28].

HDL is made up of many proteins and lipids with ApoA1 accounting for over 70% of the total protein mass [29]. ApoA1 acquires cholesterol and phospholipids (PLs) by interacting with membrane transporter ABCA1. This forms the first step of Reverse Cholesterol Transport (RCT) [30,31]. Complete deficiency of ApoA1 is very rare (<1% of the population) and presents with planar xanthomas and premature CVD [32,33]. ApoA1 variants tend to be heterozygous premature terminations, frameshifts, or amino acid substitutions in the ApoA1 amino acid sequence. Patients who have these mutations usually have low levels of HDL-C and/or ApoA1, have reduced plasma LCAT activity, and often, but not always, develop premature CVD [34]. A specific variant of ApoA1, A164S, however, is not associated with reduced HDL-C levels but is associated with increased risk of myocardial infarction, indicative of potential loss of anti-atherosclerotic function of ApoA1 with this mutation [35,36].

In contrast, those found with the Milano variant of ApoA1 exhibit no increase in intima-media thickness (IMT) compared with controls despite having low HDL-C and ApoA1 levels and hypertriglyceridemia [37]. Furthermore, treatment with a recombinant ApoA1_Milano_ was found to induce plaque regression in a rabbit model of atherosclerosis [38] while in vitro experiments using this recombinant protein also demonstrated increased CEC and enhanced antioxidant and anti-inflammatory functions compared with normal ApoA1 [39]. ApoA1_Milano_ also displayed increased anti-inflammatory activity compared to native HDL, thus stabilizing atherosclerotic plaques in a mouse model [40]. These findings are a classic example of how measures of HDL function, as opposed to HDL-C quantity, are more important as measures of CVD risk. In the case of ApoA1_milano_, the mutated protein appears to have superior function which compensates for reduced HDL-C levels in vivo. Despite the beneficial effects ApoA1 appears to illicit in regression of disease, there have been studies to show the opposite with HDL retarding the progression of atherosclerosis. Craeyveld and colleagues, using a gene transfer method in LDLr^−/−^ mice fed a Western-style diet, found that ApoA1 transfer increased collagen content in lesions but did not induce regression of atherosclerosis [41]. Similar results were seen earlier in apoE^−/−^ mice fed a Western-type diet that underwent aortic transplantation. Grafts expressed a human ApoA1 transgene and this merely deferred disease progression compared with controls [42].

CER-001, an artificial HDL mimetic composed of recombinant human ApoA1, showed promise in regressing diet-induced atherosclerosis in LDLr^−/−^ mice. Not only did CER-001 enhance cholesterol elimination, it was found to reduce vascular inflammation coincident with a 32% reduction in plaque size after multiple-dose treatment [43]. This same mimetic was brought forward for clinical trial in patients with familial hypoalphalipoproteinemia, a disease characterized by increased atherogenic burden and reductions in cholesterol removal. The study took place over 6 months with patients receiving 20 infusions of CER-001. After 9 infusions, ApoA1 and HDL-C levels had increased directly after infusion accompanied with improved cholesterol efflux, as determined ex vivo. Patients also exhibited significantly decreased mean vessel wall area as measured by magnetic resonance imaging [44]. Though this study was uncontrolled and had a very small patient cohort (*n* = 7), it served as a proof of concept in evaluating HDL infusion therapy.

While the emerging literature investigating the potential effects of HDL infusions is promising, a large limitation of these studies is the primary endpoints which focus on image-based approaches. Unfortunately, these techniques have yet to be validated adequately and are not currently used as surrogates for CVD outcome [45].

LCAT is an enzyme, synthesized in the liver, that esterifies cholesterol on lipoprotein particles [46]. Hydrophobic cholesteryl esters preferentially transfer to the inner core of the particle, unlike free cholesterol which aggregate onto the outer layer. LCAT favorably acts on HDL by interacting with ApoA1 and thus is believed to be a central part in the RCT pathway by inducing HDL maturation using cholesterol acquired from peripheral cells [47]. Those suffering from LCAT deficiency have markedly reduced HDL-C levels, yet the ramifications on CVD risk remain elusive [48]. Homozygous mutations of LCAT lead to two clinical phenotypes: familial LCAT deficiency (complete loss of function) and fish eye disease (partial loss of function). No significant difference was observed in carotid IMT of carriers of total or partial LCAT deficiency, albeit in a rather small population (*n* = 40) [49]. By contrast, another similar-sized study demonstrated that heterozygotes for LCAT gene mutations (*n* = 47) exhibit higher carotid IMT and increased CRP [50]. There are multiple potential reasons for these discrepancies, not least the small sample sizes evaluated and the great variation that occurs in measurements of IMT. LCAT deficiency is extremely rare and cases that are reported vary in lipid profiles. This makes it difficult to determine the contribution LCAT-specific deficiency has on atherosclerotic burden. GWAS analysis revealed that, independent of other lipid traits, genetic variants at the LCAT locus are strongly associated with HDL-C [23,51]. Despite this, LCAT single-nucleotide polymorphisms identified were not significantly associated with CHD [52].

While we have undoubtedly learned a tremendous amount about HDL metabolism through genetics, there remains a major disconnect between GWAS outcomes and epidemiological studies. GWAS and epidemiological findings have been replicated across multiple populations and, therefore, there is little doubting their validity. The potential that HDL-C is now merely a biomarker, and a strong biomarker at that, as opposed to playing an active role within atherogenesis has been purported. In light of these recent studies, and in conjunction with clinical trial outcomes, it has become increasingly difficult to defend the once-hailed HDL-C hypothesis. Conversely, the possibility that measurements of static HDL-C are an oversimplified gauge of HDL biology has also been suggested. In the next stage of this review we will speak to the assigned atheroprotective functions of HDL and how they are modulated under disease settings. These functions may confer more appreciation of the complexity of HDL and lead to more informative biomarkers and better disease prognosis.

## 4. Looking to the Future—HDL Functionality

**Figure 1:**
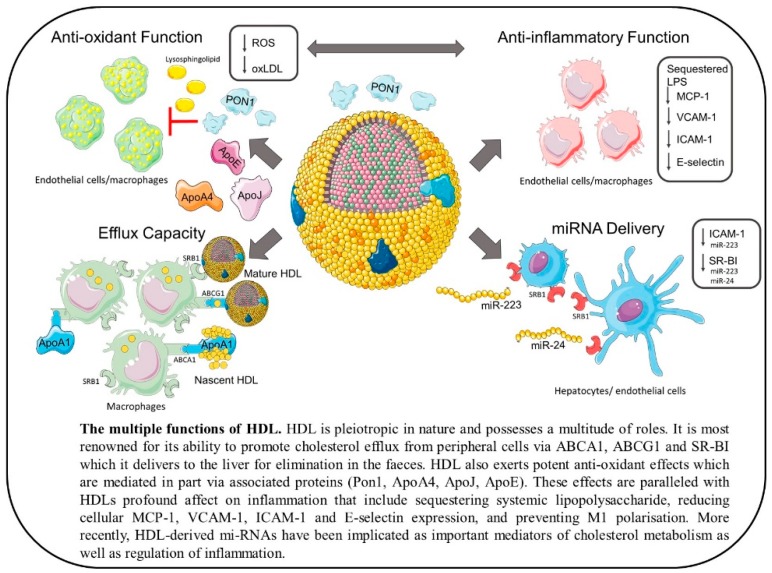
The multiple functions of HDL.

HDL particles have a myriad of functions ranging from promotion of RCT to providing anti-inflammatory and antioxidative effects as well as having anti-thrombotic and anti-apoptotic activity [53] (outlined in Figure 1). In fact, these functions are often closely linked and have been shown to correlate positively with each other [54]. As HDL is so heterogeneous it is often the subpopulations and composition of these particles that dictates their function, so high HDL-C where HDL is dysfunctional may not only have no clinical benefits but may even increase risk of CVD.

### 4.1. Cholesterol Efflux Function

Perhaps the most well-described function of HDL is its ability to accept cholesterol from peripheral cells including adipocytes, macrophages, and endothelial cells [55,56,57,58]. Cholesterol is effluxed from cells via cholesterol transporters ABCA1, ABCG1, and scavenger receptor class B type 1 (SR-B1) [58,59,60,61,62]. ABCA1 is crucial to the biogenesis of mature HDL particles and, therefore, their function. It is expressed in the liver and intestine where it both secretes ApoA1, the major protein of HDL, and effluxes cholesterol to this lipid-poor particle [63,64]. However, both adipocytes and macrophages also express ABCA1 and efflux cholesterol to lipid-poor ApoA1 via this transporter. This gives the nascent HDL particle a discoidal shape. ABCG1, on the other hand, effluxes cholesterol to mature HDL from macrophages, but not adipocytes, while mature HDL accepts cholesterol via SR-B1 from both cell types [55,58]. While macrophage cholesterol efflux contributes least to circulating HDL-C quantity, it is most important in preventing the development of atherosclerosis [65]. In fact, this is the first step of the atheroprotective pathway of macrophage-to-faeces RCT.

The role of HDL in cholesterol efflux is often measured ex vivo using HDL-, ApoA1-, or apoB-depleted serum isolated from both humans and animals as acceptors for cholesterol-loaded cells [66,67]. De la Llera-Moya et al. used this model to demonstrate that CEC is independent of HDL-C and it is in fact the concentration of the preβ-1 subpopulation of HDL that determines CEC [68]. These “cholesterol efflux assays” have also been used to show that HDL CEC is reduced during inflammation coincident with reduced preβ-1a HDL [67,69]. Consistent with this, it has been shown that preβ-1 HDL particles potently induce cholesterol efflux [70]. In 2011, a study by Khera et al. on both healthy patients and patients with CAD (*n* = 996 total) demonstrated that CEC inversely associated with both carotid IMT and CAD, independent of HDL-C [71]. In 2016, analysis on patients enrolled in the Dallas Heart Study (*n* = 1972) revealed that CEC improves risk prediction for atherosclerotic CVD [72]. Congruous with this, a meta-analysis of fifteen studies in 2017 reported an inverse association between CEC and cardiovascular risk, independent of HDL-C [73]. However, there are conflicting reports on the use of CEC to predict CVD with Kopecky et al. finding no association in patients with end-stage renal disease (ESRD) (*n* = 1147) [74] and Zimetti et al. reporting no association between CEC and atherosclerotic burden in healthy, elderly individuals (*n* = 59) despite having higher levels of CEC than a sex-matched younger cohort [75]. These findings suggest that benefits of CEC may be lost with age or in certain disease states. Noteworthily, the study by Kopecky et al. omitted healthy control patients and hence may have biased towards patients with impaired CEC, which was difficult to interpret due to lack of a comparator. Finally, the study by Zimetti et al. was much smaller in number than other studies, but the findings indicate “selection of the fittest” such that octogenarians who have survived middle age without succumbing to CVD may achieve this in part by having higher CEC. As HDL functional assays are not standardized, it is difficult to ascertain whether ex vivo experiments translate to clinically relevant endpoints given their complexity and variability.

The final function of HDL within the RCT pathway is to deliver cholesterol to the liver for excretion. HDL-C is selectively taken up by SR-B1 expressed on hepatocytes [76]. It has been shown in mice that hepatic SR-B1 deficiency results in increased HDL-C due to lack of flux through the liver [76,77] with increased cholesterol retention within the arterial wall ensuing [78]. By contrast, overexpression of hepatic SR-B1 increases cholesterol uptake and lowers HDL-C but effectively increases macrophage-to-faeces RCT [79,80]. These findings speak to the importance of the HDL flux hypothesis. Raising HDL-C levels in the absence of enhancing hepatic clearance of this cholesterol will inevitably result in futile cycling of cholesterol with atherogenic LDL particles given the continual exchange of cargo amongst lipoproteins in vivo. Enhanced flux of cholesterol through the liver into the fecal compartments indeed compensates for reduced HDL-C in the setting of SR-B1 overexpression and is arguably one of the most important steps of RCT beyond serum CEC.

While it is evident that SR-B1 is important for hepatic cholesterol clearance, its bidirectional flux capabilities within macrophages appear to suggest that net cholesterol efflux via SR-B1 is negligible compared to ABCA1 and ABCG1 [81].

### 4.2. Antioxidative Function

HDL also has potent antioxidative and anti-inflammatory properties. These can be attributed to the vast number of proteins and lipids that constitute this biomolecule. ApoA1 is multifunctional as not only can it promote cellular cholesterol efflux, but it can also act on LDL, rendering it resistant to oxidation by aortic endothelial cells. This effect was shown in LDL from both mice and humans by ApoA1-mediated removal of lipids required for oxidation [82]. In addition to this, methionine residues on ApoA1 have the capacity to reduce LDL-associated lipid hydroperoxides (LOOHs), primary products of free radicals, to inactive lipid hydroxides (LOHs) [83].

Other apolipoproteins associated with HDL, in particular, ApoE, ApoJ, and ApoA-IV, also have established antioxidative functions [84,85,86], while the antioxidative contribution of ApoA-II is unclear with pro-atherogenic effects also reported [87,88]. A reason for these observed pro-atherogenic effects could be that when ApoA-II is overexpressed it replaces paraoxonase-1 (PON-1), another important antioxidative protein that is primarily found associated with HDL [88]. PON-1 exerts antioxidative effects on macrophages as demonstrated by Rozenberg et al. in which incubation of macrophages from PON-1^−/−^ mice with human PON-1 decreased oxidative stress [89]. In addition to its protective effect in macrophages, PON-1 has been reported to hydrolyze short-chain oxidized PLs on LDL, thereby protecting against oxidized LDL (oxLDL) and atherosclerosis, as shown in mouse models both lacking and overexpressing serum PON [90,91]. In contrast to these results, Marathe et al. report that the hydrolysis of oxidized PLs by HDL is mediated by platelet-activating factor acetylhydrolase (PAF-AH) rather than PON-1 [92,93]. It is also worth noting that the role of HDL-associated vitamin E is unclear, with some data suggesting it can mediate antioxidative effects by reducing LOOHs [94] and others reporting that it is unable to protect against oxidized PLs [95,96].

The antioxidative functions of HDL can be completely independent to its role as a cholesterol acceptor. However, the two roles can also be linked: for example, binding of ApoA1 to ABCA1 or reconstituted HDL to ABCG1 and SR-B1 is shown to increase expression of the antioxidative enzyme superoxide dismutase in human-monocyte-derived macrophages while simultaneously reducing expression of Nox2 under conditions of oxidative stress [97]. HDL maintains endothelial cell function by promoting the ABCG1-dependent efflux of 7-ketocholesterol (7-KC), an oxysterol that is enriched on oxLDL. This efflux resulted in the reduction of 7-KC-mediated reactive oxygen species (ROS) production in human aortic endothelial cells (HAECs) and the rescue of eNOS activity, a marker of endothelial cell function [98]. In addition, LCAT, the main function of which is in RCT, can hydrolyze oxidized PLs, albeit probably to a lesser extent than PAF-AH [99,100].

### 4.3. Anti-Thrombotic Function

Prostacyclin (PGI2) is a powerful inhibitor of platelet activation derived from arachidonic acid that promotes smooth muscle relaxation and prevents vascular smooth muscle cell (vSMC) proliferation [101]. HDL promotes activation of PGI2 release. It does this via two known mechanisms. HDL-associated cholesterol esters serve as donors for PGI2 production by cyclooxygenase-2 (COX-2) enzymes [102,103]. Another lipid associated with HDL, sphingosine-1-phosphate (S1P), also serves as a cyclic adenosine monophosphate (cAMP) modulator, increasing its production in vSMCs, thereby inducing PGI2 production [104].

### 4.4. Anti-Inflammatory Function

Crucial to HDL’s beneficial effects are its anti-inflammatory functions which are manifold. Indeed, some of the anti-inflammatory effects of HDL are dependent on its antioxidative effects. Monocyte chemoattractant protein-1 (MCP-1) is involved in the recruitment of monocytes and is, therefore, an important part of the inflammatory component of atherosclerosis [105,106]. MCP-1 expression is induced by oxLDL and it has been demonstrated that HDL inhibits the production of MCP-1 in vSMCs. HDL also decreased production of ROS and reduced NAD(P)H-oxidase activation in vSMCs. These effects were mediated by lysosphingolipids present on the HDL particle and dependent on SR-B1 [107]. In addition to this, it has been reported by Mackness et al. that PON-1 inhibits oxLDL-induced production of MCP-1 by endothelial cells in vitro [108]. These results show that the direct antioxidative effects of HDL on oxLDL promote anti-inflammatory benefits and are integrally linked.

One of the main anti-inflammatory properties of HDL is thought to be its ability to sequester LPS in circulation and therefore prevent it from activating the toll-like receptor 4 (TLR4) signaling pathway in monocytes [109,110]. Parker et al. showed, using reconstituted HDL (rHDL), that surface PLs on the lipoprotein particle bind and sequester the LPS [111]. These effects translate from ex vivo treatments to in vivo with elevated HDL levels protecting against an LPS challenge in mice [112] and infusion of rHDL significantly reducing proinflammatory cytokine secretion induced by LPS in humans [113]. Furthermore, sepsis patients with low HDL-C have poorer prognosis compared with patients with normal HDL-C [114].

The expression of vascular cell adhesion molecule-1 (VCAM-1), intercellular adhesion molecule-1 (ICAM-1), and E-selectin by activated endothelial cells promotes the adhesion of leukocytes to the blood vessel walls. Activation of endothelial cells is regulated by the pro-inflammatory nuclear factor-kappaB (NF-κB) signaling pathway [115]. It has previously been demonstrated, however, that pre-incubation of human umbilical vein endothelial cells (HUVECs) with HDL could reduce tumor necrosis factor-alpha (TNF-α)-induced expression of VCAM-1, ICAM-1, and E-selectin [116,117]. This anti-inflammatory effect was most strongly conferred by smaller, dense HDL and was independent of ApoA1, as was established when ApoA1 was replaced by apo-AII [117].

Having migrated through the endothelial monolayer, monocytes begin to differentiate into proinflammatory M1 macrophages, accelerating atherosclerosis. Human blood monocyte differentiation into M1 macrophages ex vivo is inhibited in the presence of HDL [118]. Addition of HDL to co-cultures of human peripheral blood monocytes and activated T cells dampened the proinflammatory response of the monocytes as evidenced by decreased T-cell-induced secretion of cytokines including TNF-α, interleukin (IL)-1β, and IL-6 [119].

HDL may also provide anti-inflammatory benefits to macrophages by sequestering and reducing the bioavailability of the inflammatory protein serum amyloid A (SAA) in circulation. Data reveals that this decreases SAA-induced secretion of IL-6 and MCP-1 from macrophages as well as reducing expression of pro-inflammatory sPLA2-IIE and pro-thrombotic sPLA2-V [120,121,122]. In order to sequester SAA, however, the HDL particle must carry it in place of ApoA1, with evidence that this causes HDL to lose its anti-inflammatory effects by reduced inhibition of MCP-1 in vSMCs [123]. It has also been demonstrated ex vivo that cholesterol efflux dampens the TLR4-mediated inflammatory response of macrophages, thereby linking the cholesterol efflux function of HDL to that of its anti-inflammatory function [124,125].

### 4.5. Cellular Interactions with HDL

HDL carries out many of its functions by interacting with and binding to cellular receptors, be they macrophages, adipocytes, or endothelial cells. In terms of cholesterol efflux, HDL binds to ABCA1, ABCG1, and SR-B1. Lipid-poor ApoA1 binds directly to ABCA1 with high affinity, stabilizing the transporter and protecting it from degradation. This results in an increase of ABCA1 at the cell surface which causes the plasma membrane to bend. This creates a domain that more ApoA1 binds to in order to solubilize and accept cholesterol and PLs [126,127]. HDL binds directly to SR-B1, forming a hydrophobic channel in the plasma membrane through which cholesterol is effluxed passively down the cholesterol concentration gradient [128,129].

It is worth noting that we have outlined evidence pertaining to HDL function and positive influence on CVD risk outcomes in predominantly mouse models. While we have outlined the wide variety of beneficial functions of HDL, it is important to note that this particle is incredibly heterogeneous and inherently dynamic. Therefore, differentially modulated HDL may have compromised anti-atherogenic effects, or in some cases, pro-atherogenic effects. Therapies that raise HDL-C have failed at eliciting cardioprotection with focus now turning to HDL function as a key mediator of disease regression. HDL is an extremely complex particle with a spectrum of lipids and proteins that potentially make each particle functionally and structurally different. It is indeed plausible that therapies aimed at enhancing HDL functions may prove to have greater clinical benefit than previous efforts aimed at raising static HDL-C levels.

## 5. HDL Composition—Implications for Functionality

While HDL functions have been shown to be attenuated under acute inflammatory and chronic disease settings, the cause of this dysfunction is more difficult to establish due to the inherent complexity of HDL particles. HDL is an emulsion of lipids, proteins, metabolites, and miRs and changes in the composition of any of these individual components may impact on HDL functionality. In this review we will outline some key characteristics of HDL particle composition, which have been reviewed in depth elsewhere [130,131,132,133]. HDL has a high protein/lipid ratio in which ApoA1 predominates, making up 70% of the total protein content of the particle [134]. Apo-AII follows behind, making up 10–15% of the total protein. More than 80 other proteins and 200 lipids have been shown to be associated with HDL and make up the rest of the particle. The variety of proteins and lipids add to HDL particle diversity and dispersity, thus making it unlikely that each particle is enriched with the same spectrum of proteins and lipids [135].

## 6. The HDL Lipidome

The HDL lipidome comprises around half of the particle’s mass and includes over 200 individual species of lipid, the most abundant being PLs, sphingolipids, cholesterol, cholesteryl esters, and triglycerides which have been identified using electrospray ionization tandem mass spectrometry [136,137]. In terms of CVD prediction, cholesterol is undoubtedly the most important lipid associated with HDL. Free cholesterol accounts for 5–10% of total HDL lipid weight and is distributed throughout the surface lipid layer of the particle [133,136]. However, most of the sterol component of the lipidome consists of cholesteryl esters (30–40%) which are formed through the enzymatic reaction of LCAT with cholesterol [136,138]. Cholesteryl esters are more hydrophobic and therefore aggregate in the center of the HDL particle [138]. PLs are the major component of the HDL lipidome, making up 40–50%, and are predominantly located on the surface of HDL [136]. HDL-PL content is regulated by phospholipid transfer protein (PLTP) which transfers PLs between lipoproteins and has been shown to favor transfer between HDL subpopulations [139]. Both overexpression and knockout of PLTP have been shown to decrease HDL-C concentrations due to increased catabolism of the particle, indicating the importance of this particular type of lipid to HDL homeostasis [140,141]. Phosphatidylcholine (PC), lysophosphatidylcholine (LPC), and phosphatidylethanolamine (PE) are the most abundant of the PLs and contribute to HDL structure and fluidity of the surface lipid monolayer [133]. Minor HDL-PLs include the negatively charged phosphatidylinositol and phosphatidylserine which contribute to the surface charge of the lipoprotein and therefore play a role in HDL interactions with hepatic lipase [142]. There is evidence that the HDL lipidome has a functional role in cholesterol efflux with Yancey et al. demonstrating that PC enrichment increases SR-B1-dependent cholesterol efflux, while sphingomyelin (SM) enrichment inhibits SR-B1-dependent influx of HDL cholesterol in vitro [143]. Another HDL sphingolipid, S1P, also plays an important role in HDL function with heightened ability of HDL enriched with S1P to attenuate endothelial cell apoptosis induced by oxLDL [144]. As well as this, S1P also activates eNOS and inhibits MCP-1 expression in endothelial cells [145,146].

The HDL lipidome is significantly altered under pathophysiological and inflammatory conditions [133]. The PL component seems to be particularly affected with Papathanasiou et al. revealing that levels of PC and SM associated with HDL were significantly decreased in patients with CHD while triglycerides were increased [147]. This is consistent with the findings of Kunz et al. who observed decreased HDL-C and HDL-PLs and elevated HDL-triglycerides in CAD patients [148]. Hypertension has also been demonstrated to decrease the net PL content of HDL, albeit with raised LPC [149,150]. Despite the beneficial effects of S1P as described above, inflammatory cytokines such as TNFα and interleukin (IL)-1β increase S1P levels, which, in turn, has been reported to enhance inflammation by inducing prostaglandin E2 production and neutrophil recruitment [151,152,153]. Inflammation also results in decreased LCAT activity, thereby raising the proportion of free cholesterol to cholesterol esters on HDL and resulting in increased bioavailability of free cholesterol on the HDL surface to be taken up by peripheral cells via SR-B1 [138,154].

## 7. HDL-Associated microRNA (miR)

The recent discovery that HDL carries and delivers miR adds a novel dimension to HDL particles that, in turn, adds to their complexity [132]. How HDL carries these miRs remains to be elucidated, although binding to PC is a potential mechanism that has been suggested [155,156]. In addition, HDL-bound miR also appear to be more resistant to degradation [157]. Vickers et al. were the first to profile the miRs associated with HDL in both healthy individuals and those with dyslipidemia [155]. They elegantly demonstrated that HDL can deliver miR to recipient cells with functional targeting capabilities demonstrating a novel mechanism of HDL-to-cell communication in vivo. Remarkably, the HDL miR profile is very specific with enrichment of HDL with both miR-223 and miR-24 in patients with familial hypercholesterolemia [155]. Delivery of miR to target cells was dependent on interaction with SR-B1 and, indeed, HDL-miR from atherosclerotic individuals induced differential gene expression compared to healthy HDL-miR in cultured hepatocytes [155]. miR-223 can attenuate ICAM-1 expression within endothelial cells and is delivered to endothelial cells via HDL, suggesting that HDL-associated miR may contribute to the anti-inflammatory functions of HDL [158]. On the contrary, miR-223 has also been shown to potently repress SR-B1 expression [159] which would prevent HDL-C clearance from circulation. Similarly, miR-24 is implicated in atherogenesis by inhibiting hepatic lipid uptake from HDL-C via SR-B1 due to repression of SR-B1 [160]. Modulation of miR in pathophysiological conditions could potentially allow the miR profile of HDL to act as a novel biomarker of disease.

## 8. The HDL Proteome

It has long been known that proteins constitute a major component of HDL particles with identification of Apo-AI on HDL [161,162,163] in the late 1960s and Apo-AII soon after [163]. The true complexity of the HDL proteome has only been unveiled recently due to major technological advances in the “omics” field. Currently, HDL is considered to carry upwards of 85 proteins; however, this number is increasing with better sensitivity of proteomics platforms and different HDL isolation techniques. One of the earliest proteomics studies on HDL particles by Karlsson et al. confirmed the presence of Apo-AI, Apo-AII, Apo-M, Apo-CIII, Apo-CII, SAA-IV, SAA, Apo-CI, alpha-amylase salivary, Apo-L, and alpha-1-antitrypsin on HDL and was also one of the first studies to identify potential differences in the proteomic composition between different-sized particles [164]. This was followed soon after by the landmark study by Vaisar et al. who took a shotgun proteomics approach to identify HDL-associated proteins in control and CAD patients. This study again highlighted association of a range of apolipoproteins, LCAT, CETP, PLTP, SAA1, A2 and A4, and PON1 and PON3 on HDL particles. In addition, numerous complement proteins, serpin proteins, and acute-phase proteins were identified on the particles, many of which were enriched on HDL_3_ from patients with CAD [165]. The association of numerous acute-phase response proteins on HDL supports the central, and often underappreciated, role of HDL in the inflammatory response. Changes in HDL_3_ protein composition observed by Vaisar et al. in patients with CAD suggest an alteration in the “quality” of HDL particles in affected patients. In particular, ApoE was found to be enriched on the CAD–HDL_3_ particles. Whether such changes in HDL composition are causal or consequential of CAD remain unanswered, but it certainly questions the pharmacological strategy to raise the number of such HDL particles. Indeed, strategies aimed at restoring HDL quality, as opposed to increasing particle quantity, may have better outcomes. It is important to note that the proteins identified on HDL largely align with their assigned functions; this is indicative that (a) HDL particles have an active role to play in protecting against atherosclerosis and (b) changes in HDL composition may impact on HDL functions.

A major challenge for HDL proteomics is the lack of standardization for isolation of HDL which would facilitate more reproducible findings across research laboratories and provide consensus about HDL-associated proteins versus contaminants. That withstanding, enforced standardization may negate potential novel findings in relation to different subtypes of HDL. Ultracentrifugation (UC) is broadly used for HDL isolation; however, it is argued that the speed of centrifugation and the length involved, as well as the exposure to salts/density gradients, induce a lot of changes to HDL particles and may be detrimental to the quality of HDL-associated proteins and, in turn, particle functionality [166]. Gordon et al. by contrast, have investigated HDL protein composition after separation by fast protein liquid chromatography (FPLC) followed by a PL pull-down step to enrich for HDL [166]. The strength to this approach is the lack of salt and the shear force used during UC and the greater resolution of HDL subpopulations from three broad categories (HDL_1–3_) to approximately 16 different subpopulations. Gordon et al. have elegantly demonstrated that the composition of particles is different within these 16 subpopulations and again suggest that the subdivision into three groupings conferred by UC may be oversimplified. We have similarly used FPLC for HDL isolation prior to proteomics analysis and also observed different protein profiles between large and small HDL particles [167]. For example, PON1 associates primarily with small- to medium-sized particles [166] that are also prone to enrichment with SAA and ApoE under inflammatory settings [167]. These findings suggest that smaller particles may be more instrumental in regulation of the immune response, but this needs to be validated at a functional level.

## 9. The HDL Proteome as a Superior Biomarker of Disease

The HDL proteome is enriched with pro-inflammatory proteins in CVD [165,168] and type 2 diabetes mellitus (T2DM) [169] which may alter cardioprotective functions. We have similarly demonstrated that the HDL proteome in mice is enriched with the acute-phase proteins hemopexin, haptoglobin, and SAA in a diet-induced obese mouse model [167]. These effects could be prevented by replacement of pro-inflammatory saturated fatty acid (SFA) with inert monounsaturated fatty acid (MUFA) in an isocalorically matched obesogenic diet [167]. The association of hepatic-derived acute-phase proteins on SFA–HDL particles coincided with increased hepatic inflammation, greater hepatosteatosis, and impaired macrophage-to-faeces RCT compared to equivalently obese MUFA-fed mice. These findings indicate that measurement of the HDL proteome could provide a novel biomarker of hepatic health in an easily accessible biofluid. Changes in HDL composition within our study correlated with reduced efflux capacity of smaller HDL particles from SFA-fed mice. However, whether these findings were causative or correlative were not investigated.

## 10. The Future of HDL—Rising from the Ashes

We are at an important crossroads in the HDL field where our core understanding of HDL biology has been questioned by the lack of efficacy of HDL-C-raising therapies and lack of association between genetically defined low HDL and CAD. While many now consider HDL-C a biomarker of metabolic disease, of little functional value, it is difficult to accept this hypothesis when particle composition is analyzed. The strong alignment of HDL proteome pathways with assigned HDL functions, and the association of very specific proteins with HDL, suggests that the particles do exert critical biological functions. Furthermore, the overlap in protein composition between UC and FPLC techniques further substantiates the specificity of HDL proteins. The greater survival of sepsis patients with high HDL-C is likely attributable to the ability of HDL to bind to, sequester, and eliminate endotoxins from circulation [114]. Similarly, measuring HDL CEC is a stronger predictor of reduced CVD than static HDL-C levels [71] and HDL anti-inflammatory functions are restored in obese rats after bariatric surgery which correlates with improved endothelial function [170]. HDL also carries a specific subset of miRs, but, in particular, miR-24 and miR-223, which repress expression of the key hepatic HDL-receptor SR-B1 [159,160,171]. These findings indicate that HDL composition is specific and fit for purpose (i.e., assigned functions) with an in-built mechanism to regulate its own metabolism in the absence of cellular nuclear machinery, which is remarkable.

While we have learned a lot about the intricacies of HDL in the last 10 years, we have much more to learn and the questions that need to be addressed are extremely challenging due to the complexity of HDL. Numerous changes in HDL composition occur simultaneously within a disease setting and include changes in multiple miRs, proteins, and lipids. Untangling the contribution of individual changes in composition to alterations in particle function is a major challenge, particularly given the difficulty in manipulating expression of the cargo on HDL. Another major challenge to researchers is the lack of understanding about the functions of HDL subparticles and their relevance in disease pathogenesis. It is quite plausible that the true power of HDL, or specific HDL subpopulations, as biomarkers of disease has not yet been realized. Much as HDL CEC is a stronger indicator of CVD than HDL-C, it is likely that HDL lipidomic/proteomic or miR composition will yield far more information as to disease prognosis than static HDL-C levels. Given our greater insight into the complexity of HDL, and the refocus from quantity to quality, we project that the future will see HDL rising like a phoenix from the ashes after a turbulent decade.

## Abbreviations

HDL	High-density lipoprotein
CVD	Cardiovascular disease
CEC	Cholesterol efflux capacity
HDL-C	High-density lipoprotein cholesterol
miR	microRNA
ApoA1	Apolipoprotein-A1
SR-B1	Scavenger receptor class B type 1
ABCA1	ATP-binding cassette transporter A1
ABCG1	ATP-binding cassette transporter G1
CHD	Coronary heart disease
CETP	Cholesteryl ester transfer protein
LDL	Low-density lipoprotein
LDL-C	Low-density lipoprotein cholesterol
CRP	C-reactive protein
MI	Myocardial infarction
GWAS	Genome-wide association studies
LCAT	Lecithin–cholesterol acyltransferase
ROS	Reactive oxygen species
RCT	Reverse cholesterol transport
IMT	Intimal–media thickness
SNPs	Single-nucleotide polymorphisms
CAD	Coronary artery disease
PON-1	Paraoxonase-1
oxLDL	Oxidized LDL
PL	Phospholipid
HAECS	Human aortic endothelial cells
vSMC	Vascular smooth muscle cells
MCP-1	Monocyte chemoattractant protein-1
VCAM-1	Vascular cell adhesion molecule-1
ICAM-1	Intercellular adhesion molecule-1
SAA	Serum amyloid A
PLTP	Phospholipid transfer protein
T2DM	Type 2 diabetes mellitus
